# Durable Response of Dabrafenib, Trametinib, and Capmatinib in an NSCLC Patient With Co-Existing BRAF-KIAA1549 Fusion and MET Amplification: A Case Report

**DOI:** 10.3389/fonc.2022.838798

**Published:** 2022-03-18

**Authors:** Yun-Tse Chou, Chien-Chung Lin, Chung-Ta Lee, Dean C. Pavlick, Po-Lan Su

**Affiliations:** ^1^ Department of Internal Medicine, National Cheng Kung University Hospital, College of Medicine, National Cheng Kung University, Tainan, Taiwan; ^2^ Institute of Clinical Medicine, National Cheng Kung University Hospital, College of Medicine, National Cheng Kung University, Tainan, Taiwan; ^3^ Department of Biochemistry and Molecular Biology, College of Medicine, National Cheng Kung University, Tainan, Taiwan; ^4^ Department of Pathology, National Cheng Kung University Hospital, College of Medicine, National Cheng Kung University, Tainan, Taiwan; ^5^ Research and Development, Foundation Medicine, Inc., Cambridge, MA, United States

**Keywords:** *BRAF* fusion, *MET* amplification, case report, double driver mutation, combination therapy

## Abstract

*BRAF* fusions are rare driver oncogenes in non-small cell lung cancer (NSCLC). Similar with *BRAF* V600E mutation, it could also activate the MAPK signaling pathway. There are a few case reports which had indicated the potential response to BRAF inhibitors and its important role as *de novo* driver mutation. In addition, the co-occurring *MET* amplification has been defined as a poor prognostic factor in patients with epidermal growth factor receptor (*EGFR*) mutant NSCLC. Currently, there are ongoing clinical trials which investigate the *MET* amplification as a therapeutic target in patients with *EGFR* mutant NSCLC and acquired resistance to osimertinib, which imply that the *MET* amplification also had a therapeutic significance. However, the co-occurring *MET* amplification had not been studied in patients with *BRAF* fusion before. A 67-year-old man was diagnosed with metastatic poorly-differentiated adenocarcinoma. He received first-line therapy with the combination of pembrolizumab and chemotherapy because the genomic test revealed wild-type *EGFR*, and negativity of ALK and ROS1 by immunohistochemical stain. Upon disease progression, the next-generation sequencing revealed co-occurring *KIAA1549-BRAF* fusion and *MET* amplification. Subsequent dabrafenib, trametinib, and capmatinib combination therapy showed a remarkable treatment effect. The combination therapy targeting the co-occurring driver mutations is a potential effective treatment for NSCLC patients. Further prospective study is still warranted to investigate the role of co-occurring driver mutations and the relevant treatment strategy.

## Introduction

*BRAF* fusions are mostly detected in melanoma, thyroid cancer, and astrocytoma and are rare driver oncogenes in non-small cell lung cancer (NSCLC) (1). They present in approximately 0.2% of NSCLC patients and have different activation mechanisms with *BRAF* mutations (2). The *KIAA1549-BRAF* fusion will cause constitutive activating kinase activity resulting from the loss of the BRAF autoregulatory N-terminal domain and retention of the C-terminal kinase domain (2). Currently, there is no prospective clinical trial regarding the treatment strategies for patients with *BRAF* fusion and no US Food and Drug Administration approved therapy. However, there are a few case reports have demonstrated the potential response to monotherapy with the BRAF inhibitor vemurafenib or the MEK inhibitor trametinib (3, 4), which indicate that the *BRAF* fusion is also an important targetable driver mutation.

In addition, the role of co-occurring *MET* amplification had been widely studied in patients with epidermal growth factor receptor (*EGFR*) mutant NSCLC and was defined as a poor prognostic factor (5). The combination of capmatinib and osimertinib could provide better progression-free survival than chemotherapy in patients with osimertinib-resistant *EGFR* mutant NSCLC and *MET* amplification (6). There are also ongoing clinical trials investigating the role of combination therapy targeting *MET* amplification in *EGFR* mutant NSCLC and acquired resistance to osimertinib (7, 8), which indicates the *MET* amplification had therapeutic role and combination of targeted therapy is a potential therapeutic strategy. However, the role of co-occurring *MET* amplification and the relevant treatment strategy has not been studied in patients with *BRAF* fusion. The profile of adverse events when using combination therapy was not assessed before. This case report presents co-occurring *KIAA1549-BRAF* fusion and *MET* amplification and showed a durable response and tolerant adverse events to combination therapy with dabrafenib, trametinib, and capmatinib.

## Case Presentation

In December 2018, a 67-year-old man was diagnosed with stage IV pulmonary poorly differentiated adenocarcinoma with negativity of TTF-1 and P40 expression (**Supplementary Figure 1A**). The tumor involved the right middle lobe and had multiple satellite masses and pleural involvement. The *EGFR* mutation test yielded no sensitizing mutations, and the immunohistochemical (IHC) staining for anaplastic lymphoma kinase (ALK) and ROS proto-oncogene 1 (ROS1) were both negative. The IHC staining of programmed death-ligand 1 was 3%. The next generation sequencing (NGS) could not be performed due to insufficient tissue. Thus, the combination of cisplatin, pemetrexed, and pembrolizumab was administered. After 12 months, the patient demonstrated disease progression with an enlarged right middle lung mass. The NGS was suggested in order to optimize subsequent therapy. To obtain sufficient tissue for NGS, video-assisted thoracoscopic surgery (VATS) biopsy was performed due to previous experience of insufficient tissue from computed tomography-guided biopsy.

**Figure 1:**
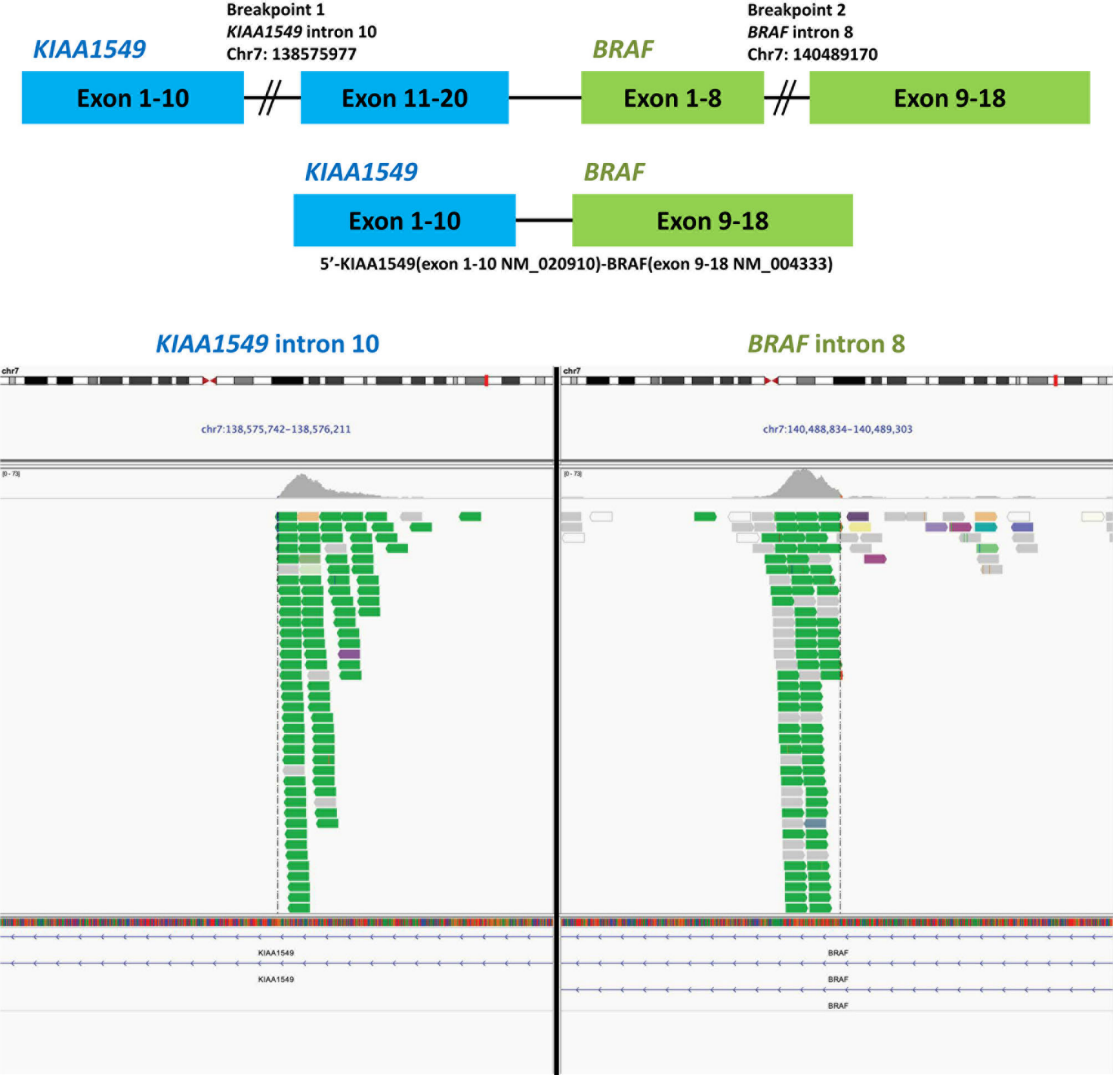
Next-generation sequencing of the tissue specimen from the video-assisted thoracic surgery biopsy of right middle lung cancer revealed a *BRAF-KIAA1549* fusion. The data was provided by the Department of Research and Development, Foundation Medicine Inc.

The pathologic report of VATS biopsy revealed also poorly-differentiated adenocarcinoma, and the tumor cells were also negative for TTF-1 and P40 (**Supplementary Figure 1B**). Furthermore, NGS by FoundationOne^®^ CDx revealed *KIAA1549-BRAF* fusion (**Figure 1**) and *MET* amplification (copy number gain: 10; **Table 1**). The detailed report was summarized in **Supplementary Figure 2A**. The patient then received second-line chemotherapy with docetaxel and ramucirumab. However, after approximately 8 months, he experienced disease progression with increased right pleural effusion. With multidisciplinary team discussion, capmatinib was administered as the third-line therapy to target the *MET* amplification according to the GEOMETRY mono-1 study (9). Unfortunately, after 3 months of therapy with capmatinib, the right pleural effusion increased gradually concurrently with development of left pleural effusion. A pleuroscopic biopsy was performed on the left pleura and also revealed poorly-differentiated adenocarcinoma with negative TTF-1 and P40 expression on tumor cells (**Supplementary Figure 1C**). To optimize the subsequent treatment strategy, NGS was repeated and revealed *KIAA1549-BRAF* fusion and absence of *MET* amplification (**Table 1**). The detailed report was summarized in **Supplementary Figure 2B**. Based on the second NGS report and previous case report which indicate BRAF inhibitor could be a potential therapy for patient with *BRAF* fusion (4), we shifted the treatment to dabrafenib and trametinib.

**Table 1:** The detailed genomic alterations of the repeated biopsy specimen detected by FoundationOne CDx.

VATS biopsy on Dec 2019	Pleuroscopic biopsy on Nov 2020
*BRAF-KIAA1549* fusion	*BRAF-KIAA1549* fusion
*MET* amplification^†^	*BCL2* P59L
*CDK6* amplification	*SMARCA4* Q356
*BCL2* P59L^††^	*TP53* C275
*NOTCH1* V1575L^††^	
*TP53* C275	

^†^Copy number gain 10.

^††^subclone.

VATS, video-assisted thoracic surgery.

**Figure 2:**
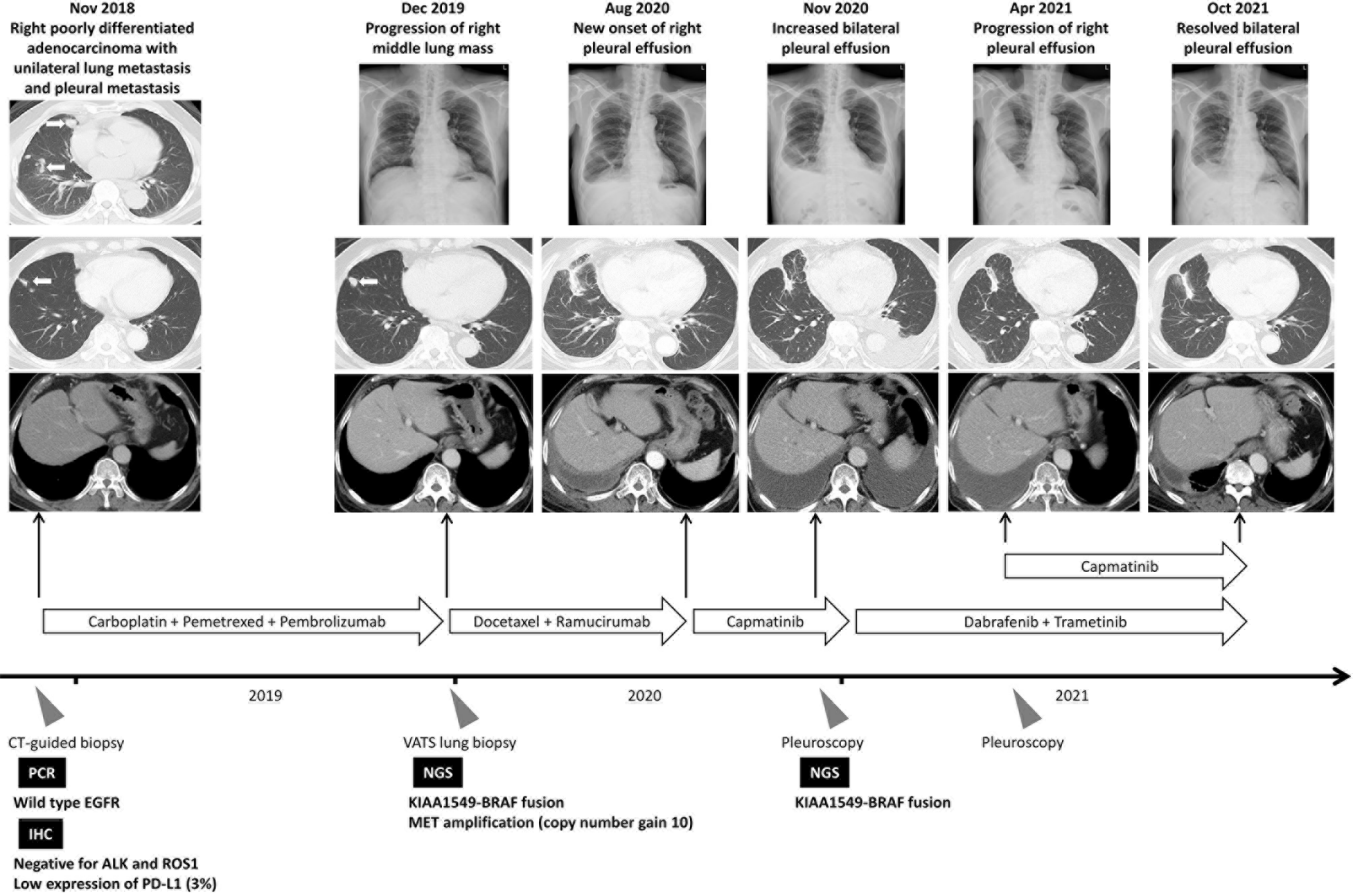
Summary of treatment courses mentioned in this case report. The white arrows indicate the target lesion in the serial image study. *ALK*, anaplastic lymphoma kinase; CT, computed tomography; *EGFR*, epidermal growth factor receptor; IHC, immunohistochemical; NGS, next-generation sequencing; PCR, polymerase chain reaction; *ROS1*, ROS proto-Oncogene 1; VATS, video-assisted thoracic surgery.

However, the patient suffered from progressive exertional dyspnea after 3-month treatment of dabrafenib and trametinib. The plain film radiograph revealed resolved left pleural effusion but had a drastically increased right pleural effusion. Repeated pleuroscopic biopsy at right side pleura still revealed poorly-differentiated adenocarcinoma with negativity in TTF-1 and P40 expression (**Supplementary Figure 1D**). The pleurodesis was performed but invalid because of multiloculated pleural effusion. We had also tried to performed NGS analysis but failed due to low tumor purity. Nonetheless, the resolved left pleural effusion and drastic increase of right pleural effusion, which developed immediately after discontinuation of capmatinib, implied that the tumor in right side pleura still harbor co-occurring *MET* amplification and *KIAA1549-BRAF* fusion while the tumor in left side pleura harbor only *KIAA1549-BRAF* fusion. In addition, the performance status of the patient was poor and chemotherapy could not be administrated. Based on the successful combination strategy targeting *MET* amplification in *EGFR* mutant NSCLC (6) and the animal study demonstrated the potential benefit of combination therapy in *BRAF* mutation (10), the treatment strategy was shifted to combination therapy with dabrafenib, trametinib, and capmatinib after multidisciplinary team discussion. Finally, the right pleural effusion subsided and remained stable for more than 6 months. The treatment course is summarized in **Figure 2**. During combination therapy, higher grade of adverse events developed compared to monotherapy, including peripheral edema, nausea, fatigue, skin rash, and fever. The symptom got subsided partially after the reduction of capmatinib dosage to 100mg twice daily. The profile of adverse events and the doses of relevant targeted therapy was summarized in **Table 2**.

**Table 2:** The toxicity profile of the combination therapy of dabrafenib, trametinib, and capmatinib.

Targeted therapy	Adverse event	Grade
**Capmatinib 200mg twice daily**	Peripheral edema	1
	Nausea	1
**Dabrafenib 150mg twice daily**	Fever	1
**Trametinib 2mg once daily**		
**Dabrafenib 150mg twice daily**	Peripheral edema	3
**Trametinib 2mg once daily**	Nausea	1
**Capmatinib 100mg twice daily**	Fatigue	2
	Skin rash	1
	Fever	1

## Discussion

*BRAF* fusions are rare driver oncogene in patients with advanced NSCLC (2), which mostly be discovered as acquired resistance mechanism to EGFR-TKIs and rarely be a *de novo* mutation (1, 11). They lack the RAS-binding auto-inhibitory domain found in the N-terminal and the fusion partner often harbors a constitutive dimerization or oligomerization motif (2). Similar with *BRAF* V600E mutation, the *BRAF* fusion could also activate the mitogen-activated protein kinase (MAPK) signaling pathway and respond to MEK inhibitor in a case with melanoma (12). Similarly, there are also case reports which had demonstrated potential therapies in NSCLC patients with *BRAF* fusion. For example, a patient with advanced lung adenocarcinoma harboring the *LIMD1-BRAF* fusion showed a partial response and remained on treatment with trametinib for more than 7 months (3). Another case report on a patient harboring the *TRIM24-BRAF* fusion demonstrated a durable response to vemurafenib (4). The patient in this case study demonstrated significant decrease of the pleural effusion on the left side after receiving dabrafenib and trametinib, which aligns with the NGS report of the left side pleuroscopic biopsy (**Table 1**) and indicate the importance of *BRAF* fusion as a *de novo* driver mutation.

Combination therapy targeting co-occurring *MET* amplification has been studied in patients with *EGFR* mutations (13). In a xenograft study using osimertinib resistant *EGFR* mutant lung cancer cells with *MET* amplification, the knockdown MET signal pathway or the combination of MET inhibitor could induce tumor shrinkage, indicating that targeting *MET* amplification may reverse EGFR-TKI resistance (14). In the phase 2 INSIGHT study, which enrolled patients with *EGFR* mutant NSCLC who had *MET* amplification or MET overexpression after acquired resistance to EGFR-TKI, the median progression-free survival was 16.6 months among patients received tepotinib and gefitinib combination (6). In the exploratory analysis of phase 2 study regarding the treatment efficacy of combined erlotinib and emibetuzumab, MET inhibitor patients with a high level of MET expression (MET immunohistochemistry score of 3+ in at least 90% of tumor cells) had significantly long progression-free survival when receiving combination therapy with erlotinib and emibetuzumab (20.7 versus 5.4 months, hazard ratio 0.39 [0.17–0.91]) (5). Currently, there are also ongoing clinical trials investigating the role of combination with capmatinib therapy in osimertinib-resistant *EGFR* mutant NSCLC and *MET* amplification, including SAVANNAH (NCT03778229) (7), INSIGHT 2 (NCT03940703) (8), and GEOMETRY-E (NCT04816214). The studies above implied the clinical benefit of combination therapy to co-occurring *MET* amplification.

**Figure 3:**
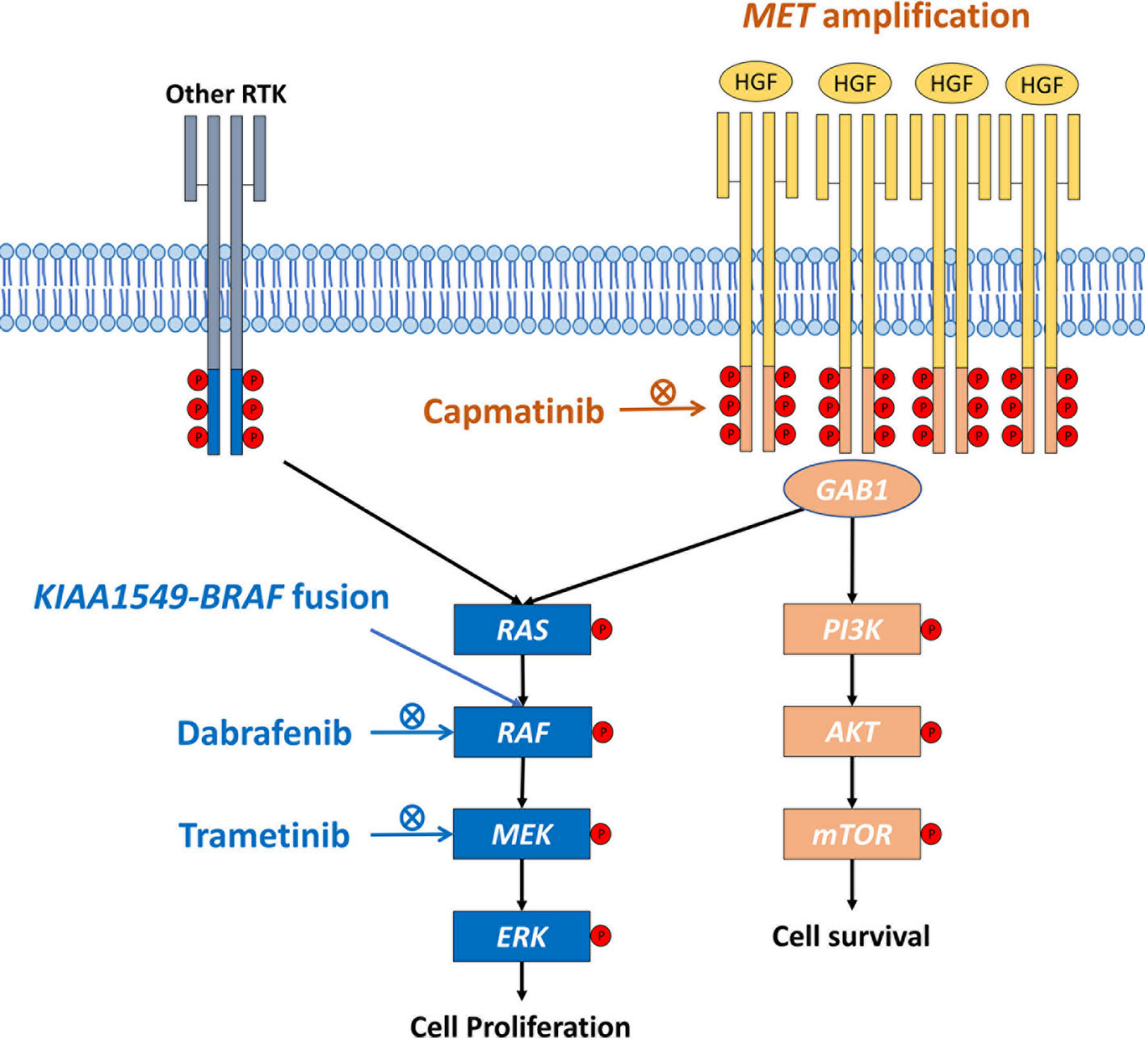
Schematic diagram of the cross-reactivity of BRAF fusion and MET amplification. HGF, hepatocyte growth factor; RTK, receptor tyrosine kinase.

Similarly, in a cell line study using primary culture from patients harboring *BRAF* mutation and *MET* amplification, the inhibition of MEK expression induces dose-responsive MET activation (10). The combination of MEK inhibitor and MET monoclonal antibody provided significant tumor shrinkage in a patient-derived xenograft mouse model of cancer with co-occurring *BRAF* mutation and *MET* amplification (10), which is in line with our case report. This study highlights the importance of combination therapy. The underlying mechanism may result from the activation of downstream MAPK pathway induced by *MET* amplification, which enhance the kinase activity of *BRAF* fusion protein. The combination therapy which targets both *MET* amplification and *BRAF* fusion is the potential treatment strategy (**Figure 3**). Here, the patient with co-occurring *KIAA1549-BRAF* fusion and *MET* amplification suffered from progressively increased pleural effusion on the right side despite receiving combination therapy with dabrafenib and trametinib. The pleural effusion resolved gradually and remained stable for more than 6 months after adding capmatinib, a selective MET inhibitor. This case report highlights the importance of comprehensive genomic profiling to identify the druggable driver mutations and the combination therapy may be a potential strategy for patients with co-occurring mutations.

Previous study regarding the adverse events in the combination therapy of different targeted therapies remains limited. According to the phase 2 study investigating the treatment efficacy of dabrafenib and trametinib in patients with *BRAF* V600E positive NSCLC, the most common grade 3 or 4 adverse events were pyrexia (11%), elevated liver enzyme (11%), hypertension (11%), and vomiting (8%) (15). Meanwhile, patients received capmatinib had adverse events of peripheral edema (51%) and nausea (45%), but these events were mostly of grade 1 or 2 (9). In this case report, patient suffered from higher grade of adverse events after combination therapy. The symptom subsided partially after the reduction of capmatinib dosage and the clinical condition remains stable. It implies that the combination therapy may aggravate the adverse event of each targeted therapy, and dose reduction instead of interruption might be a better choice.

## Conclusion

We report a case of advanced NSCLC harboring co-occurring *KIAA1549-BRAF* fusion and *MET* amplification in a patient with a durable response and tolerant adverse event to combination therapy with dabrafenib, trametinib, and capmatinib. Future prospective studies are warranted to validate the efficacy of combination therapy in patients with multiple driver oncogenes.

## Data Availability Statement

The raw data supporting the conclusions of this article will be made available by the authors, without undue reservation.

## Ethics Statement

The studies involving human participants were reviewed and approved by The Review Board and Ethics Committee of National Cheng Kung University Hospital. The patients/participants provided their written informed consent to participate in this study.

## Author Contributions

Y-TC and P-LS had full access to data in this case report and takes responsibility for the integrity and accuracy of data analysis. Y-TC and P-LS contributed to pleuroscopic examination. C-CL, DP, and P-LS contributed to the scientific review and final approval of this manuscript. All authors read and approved the final manuscript.

## Funding

The present study was funded by grant no. MOST 110-2314-B-006-102 from the Ministry of Science and Technology, Taiwan.

## Conflict of Interest

DP is an employee of Foundation Medicine Inc. (FMI) and has equity interest in F. Hoffmann-La Roche AG, of which FMI is a wholly owned subsidiary.

The remaining authors declare that the research was conducted in the absence of any commercial or financial relationships that could be construed as a potential conflict of interest.

## Publisher’s Note

All claims expressed in this article are solely those of the authors and do not necessarily represent those of their affiliated organizations, or those of the publisher, the editors and the reviewers. Any product that may be evaluated in this article, or claim that may be made by its manufacturer, is not guaranteed or endorsed by the publisher.
