# Supplementary figures and images for: Durable Response of Dabrafenib, Trametinib, and Capmatinib in an NSCLC Patient With Co-Existing BRAF-KIAA1549 Fusion and MET Amplification: A Case Report
Source: Front Oncol. 2022 Mar 18;12:838798. doi: 10.3389/fonc.2022.838798 (PMC8972191; doi:10.3389/fonc.2022.838798)

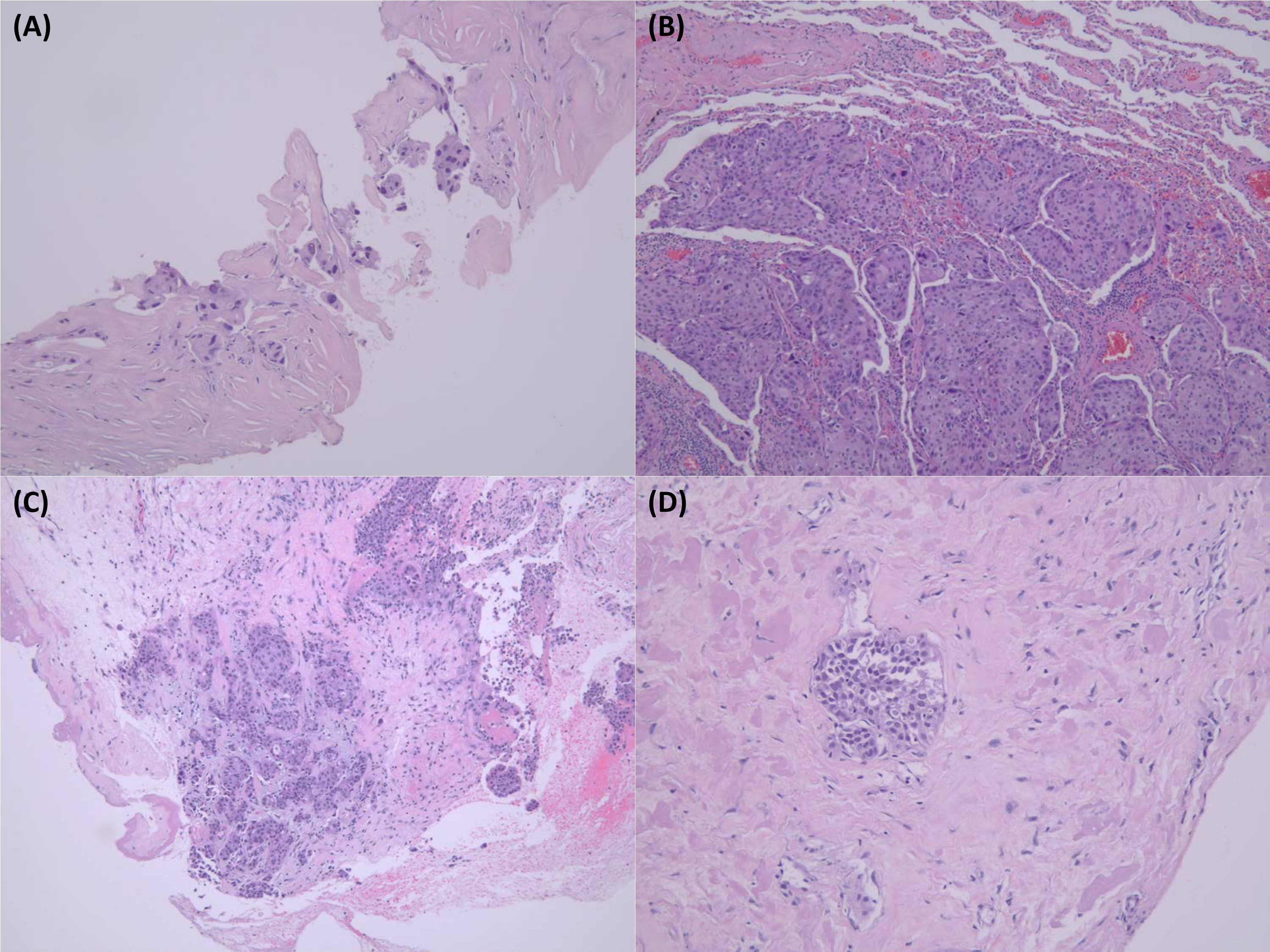

Supplement: Supplementary file 1 [file Image_1.tif]

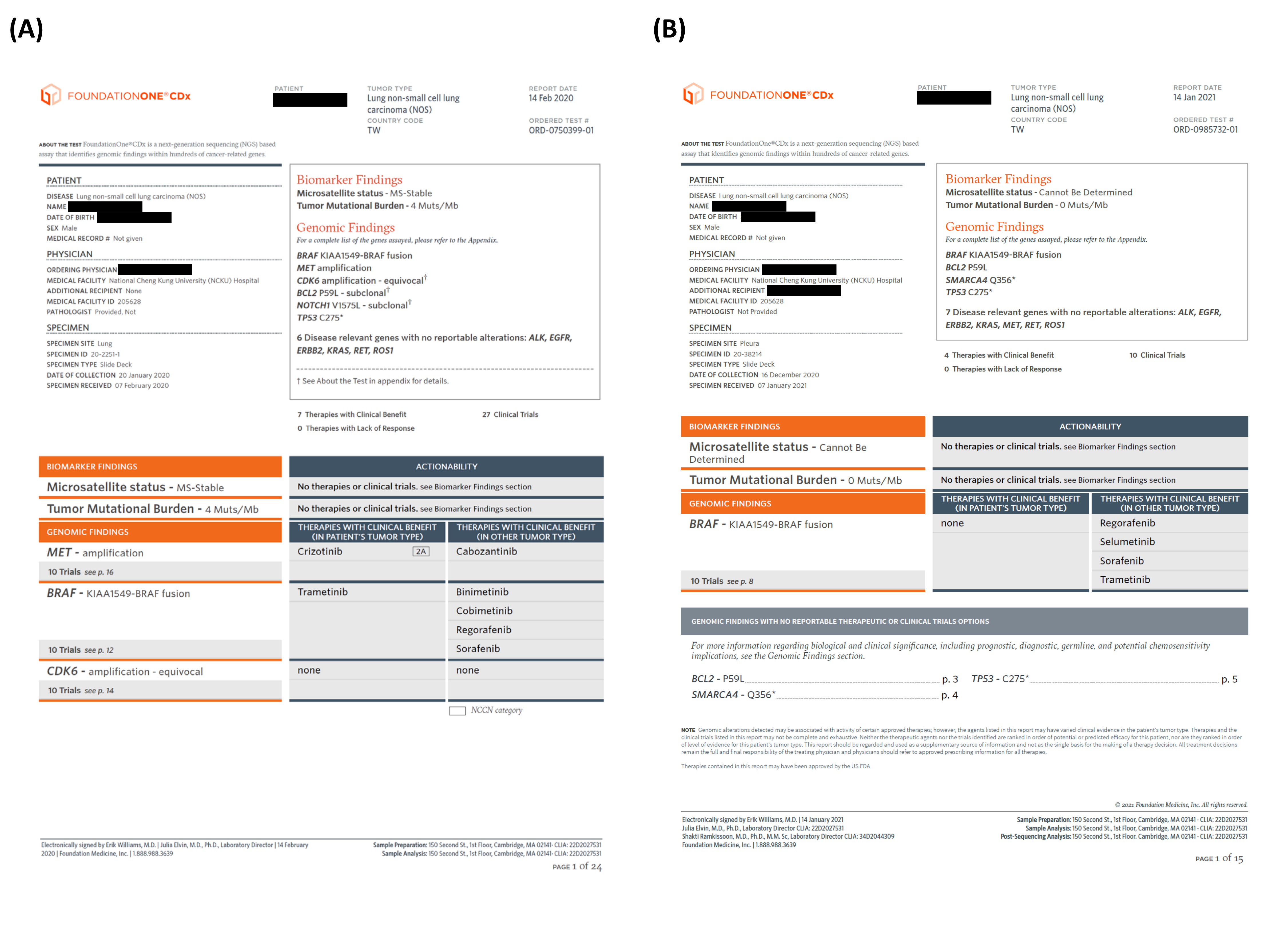

Supplement: Supplementary file 2 [file Image_2.tif]
